# A Case of Malignant Melanoma Metastasis in the Ileal Conduit

**DOI:** 10.1002/iju5.70073

**Published:** 2025-07-10

**Authors:** Noritoshi Shamoto, Yushi Naito, Yuta Sano, Kazuna Matsuo, Satoshi Inoue, Tomoyasu Sano, Tomokazu Kimura, Shohei Ishida, Yoshihisa Matsukawa, Shusuke Akamatsu

**Affiliations:** ^1^ Department of Urology Nagoya University Graduate School of Medicine Nagoya Japan

**Keywords:** bladder cancer, histopathology, ileal conduit, melanoma, metastasis

## Abstract

**Introduction:**

Tumor development in the ileal conduit is rare. Herein, we present a case of metastatic malignant melanoma occurring within the ileal conduit 17 years after radical cystectomy.

**Case Presentation:**

A 76‐year‐old Japanese man, with a history of bladder cancer treated with radical cystectomy and ileal conduit diversion, presented with gross hematuria. He had malignant melanoma diagnosed 10 years prior, with recurrent metastases managed through surgery and adjuvant therapy. During his recent admission, metastases to the subcutaneous tissue and retroperitoneum were noted. Contrast‐enhanced computed tomography revealed bilateral hydronephrosis and a tumor in the ileal conduit. Endoscopic resection confirmed metastatic malignant melanoma. The patient's renal function stabilized postoperatively, and hematuria was controlled. A palliative care approach was adopted to treat the melanoma.

**Conclusion:**

This rare case of metastatic malignant melanoma within an ileal conduit highlights the importance of histopathological examinations in patients with overlapping malignancies.

**Trial Registration:** 2016‐0474

**Summary**

- We report a rare case of metastatic malignant melanoma developing within an ileal conduit created following radical cystectomy.
- We also perform a brief literature review on tumors arising in the ileal conduit, offering insights into the histopathological analysis for the diagnosis and management of such lesions.

## 1 Introduction

Ileal conduit diversion is a common method used following radical cystectomy for bladder cancer. The ileal conduit primarily serves as a passage for urine, and tumor development within the ileal segment is rare. Malignant melanoma, a highly aggressive tumor known for its hematogenous spread, frequently metastasizes to various organs, including the gastrointestinal tract. However, ileal conduit metastasis is uncommon.

In the present report, we describe a rare case of metastatic malignant melanoma manifesting as a tumor within the ileal conduit in a patient with a history of bladder cancer and disseminated melanoma.

## 2 Case Presentation

**FIGURE 1:**
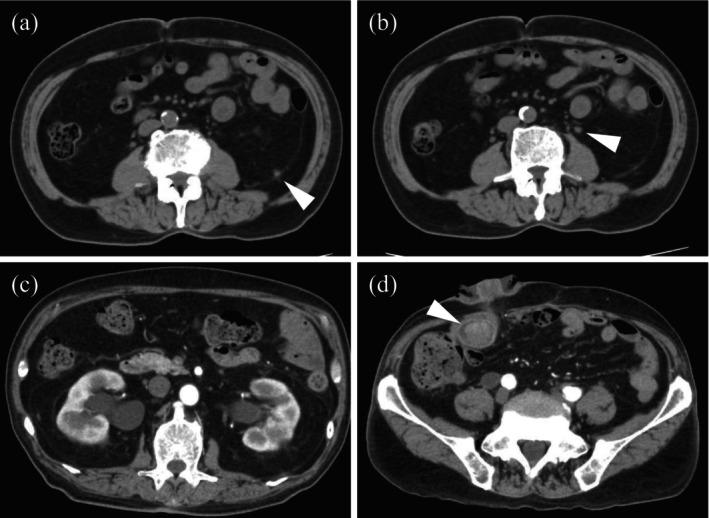
Horizontal view of computed tomography images. (a, b) Axial computed tomography scan shows two of multiple metastatic lesions (arrowhead) located in the retroperitoneum. (c) Contrast‐enhanced computed tomography shows bilateral hydronephrosis. (d) A mass (arrowhead) is visualized within the ileal conduit.

A 76‐year‐old Japanese man presented with gross hematuria. He had undergone radical cystectomy and ileal conduit diversion for urothelial carcinoma 17 years prior. His medical history included diabetes mellitus, hypertension, hyperlipidemia, myelodysplastic syndrome, and malignant melanoma. Melanoma was initially detected as a nodule on the right elbow 10 years prior to presentation. Recurrent metastases in his right upper arm were observed 6 years ago, requiring repeated surgeries and adjuvant therapy. Six months prior to presentation, numerous metastases involving subcutaneous nodules beyond the arm and retroperitoneum were observed (Figure 1a,b).

**FIGURE 2:**
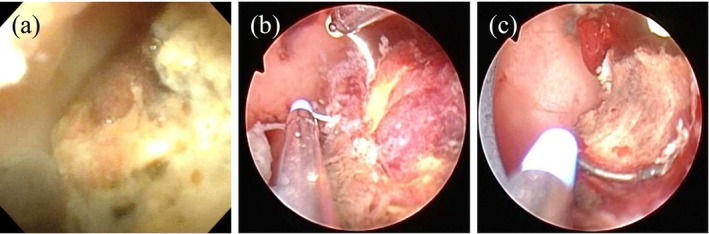
Endoscopic findings of the ileal conduit. Endoscopic images of the ileal conduit obtained before surgery (a) and during tumor resection (b, c).

At the time of admission, worsening hydronephrosis had led to pyelonephritis. Contrast‐enhanced computed tomography revealed bilateral hydronephrosis and a 2 cm mass located 10 cm away from the stoma (Figure 1c,d). Fluoroscopy‐assisted evaluation of the conduit morphology was performed. A ureteral stent was successfully placed on the right side, but placement on the left side was unsuccessful. An endoscopic examination revealed irregular ridges in the conduit (Figure 2a). After a 2‐week course of antibiotics, tumor resection was performed using endoscopic resection in saline for histopathological diagnosis (Figure 2b,c).

**FIGURE 3:**
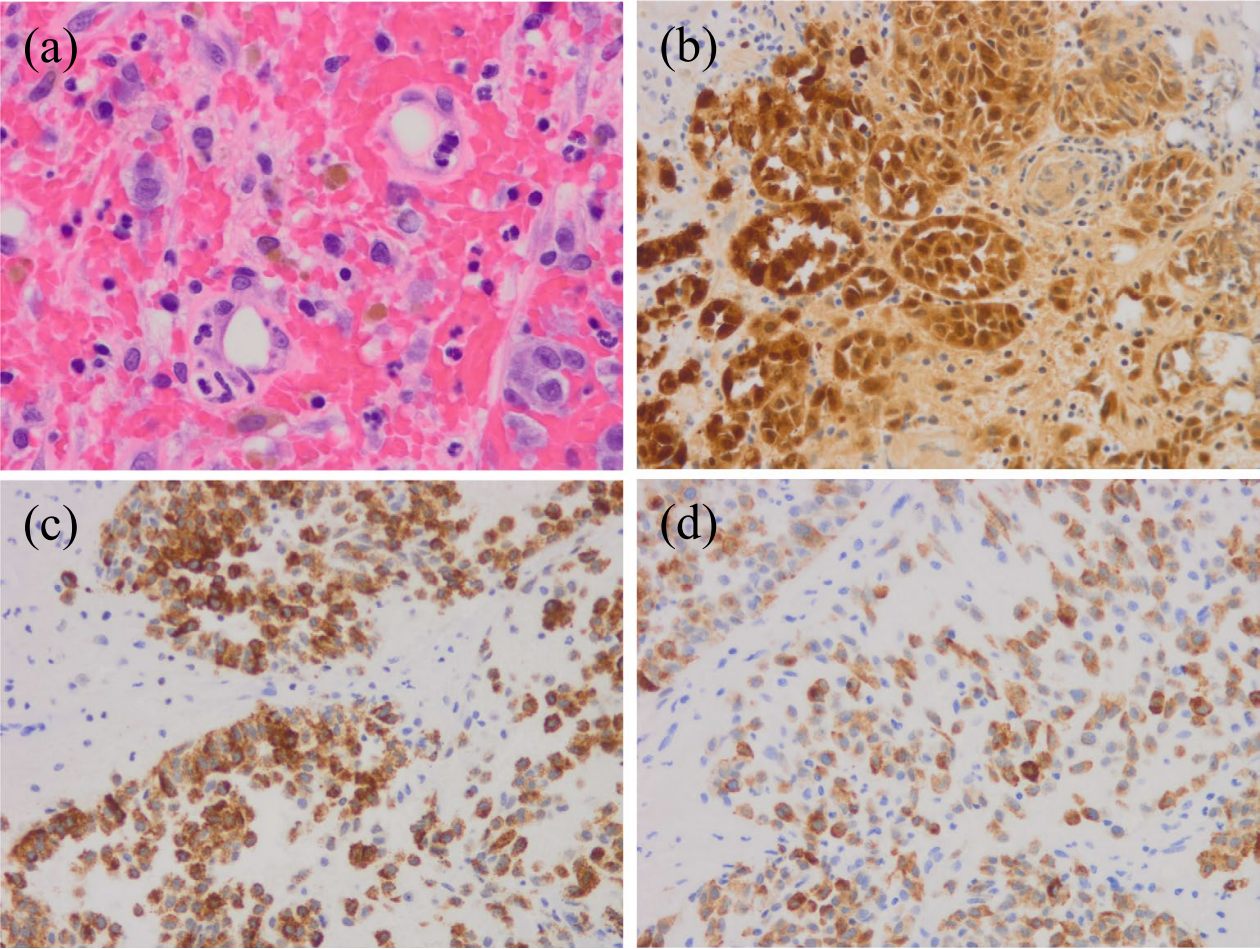
Histological assessment. (a) Hematoxylin and eosin staining (high‐power field, original magnification ×400) shows enlarged nuclei and increased chromatin; (b–d) Immunohistochemical staining (high‐power field, original magnification ×200) reveals tumor cells positive for S‐100 (b), HMB‐45 (c), and MART‐1 (d).

Histopathological analysis revealed nests of infiltrating atypical cells with enlarged nuclei and increased chromatin content. Immunohistochemical staining was positive for S‐100, HMB‐45, and MART‐1, confirming the diagnosis of metastatic malignant melanoma (Figure 3).

His renal function stabilized postoperatively, and his hematuria resolved. However, 2 months following the surgery, the patient developed gastrointestinal bleeding, requiring a blood transfusion, which led to the diagnosis of duodenal metastasis. At that point, treatment for malignant melanoma was discontinued, and the patient was transitioned to palliative care.

## 3 Discussion

We report the case of a patient with a long‐standing history of bladder cancer who remained recurrence‐free for 17 years postoperatively. Three types of malignant tumors can emerge in the ileal conduit after urinary tract diversion: disseminated metastasis of urothelial carcinoma, primary tumors originating from the ileal conduit, and metastatic lesions originating from other primary cancers. Table 1 summarizes the characteristics of these three tumor types.

**TABLE 1:** Characteristics of three types of malignant tumors in the ileal conduit.

	Disseminated metastasis of urothelial carcinoma	Primary tumors originating from the ileal conduit itself	Metastatic lesions originating from other primary cancers
Features	An average of 3.2 years postsurgery to emerge [1] Upper urinary tract recurrence is frequently observed [1]	Median interval of 17 years post‐surgery to emerge [2] Small‐intestinal neoplasms are relatively rare [3]	Time to emerge varies The causes of small‐intestinal metastases are Lung cancer, malignant melanoma, and esophageal cancer [4]

The first category involves the metastatic dissemination of urothelial carcinoma. In a review of 12 cases, Sengiku et al. [1] reported that urothelial carcinoma arose in the ileal conduit at an average of 3.2 years postsurgery. Seventy‐five percent of these cases present with upper urinary tract recurrence, with approximately half occurring at the ureter‐conduit anastomosis.

The second category encompasses tumors of ileal origin. Small‐intestinal neoplasms are relatively rare and account for only 1%–2% of all gastrointestinal malignancies, partly owing to low intraluminal stagnation and a relatively sparse bacterial load. Among these, adenocarcinoma is the most frequently encountered malignancy in the ileal conduits [3]. Although the precise mechanism of carcinogenesis remains unclear, the formation of nitrosamines through the interaction of feces and urine, as well as chronic inflammatory changes at anastomotic sites between distinct mucosal types, has been implicated [5]. In previous reports, the most common location for these tumors was the ureter‐conduit anastomosis, with a median interval of 17 years between the initial surgery and tumor development [2, 6].

The third category includes metastatic lesions from other primary cancers. Lung cancer is the most frequent cause of small intestinal metastases, followed by malignant melanoma (15%–30% of metastatic small intestinal tumors) and esophageal cancer [4, 7]. As melanoma readily disseminates through hematogenous pathways, it exhibits a pronounced predilection for the gastrointestinal tract, especially the small intestine, which has a rich blood supply [8]. Furthermore, more than half of the patients who die of disseminated melanoma demonstrate metastatic involvement in the gastrointestinal tract [9].

In the present case, the patient subsequently presented with numerous subcutaneous nodules and retroperitoneal metastases suggestive of disseminated melanoma. Considering the characteristics of the three potential tumor types in the ileal conduit, the tumor could have represented either a carcinoma arising within the conduit or hematogenous metastasis from malignant melanoma. Ultimately, pathological examination confirmed that the lesion consisted of metastatic malignant melanoma cells. This case highlights the importance of a thorough differential diagnosis in patients with atypical lesions in the ileal conduits following urinary tract diversion. Small intestinal metastases from malignant melanoma are associated with a poor prognosis. In the present case, duodenal metastasis was observed following the diagnosis of metastasis to the ileal conduit, and the patient subsequently experienced a poor clinical outcome [10].

This report has some limitations. First, it describes a single case; therefore, its findings may not be generalizable to other patients. Additionally, owing to the retrospective nature of this case, optimal management strategies for similar cases remain uncertain. Future case series and larger studies are warranted to improve our understanding of this condition.

## 4 Conclusion

We report a rare case of metastatic malignant melanoma that presented as a tumor within the ileal conduit 17 years after radical cystectomy. In patients with multiple malignancies, the histopathological analysis of newly detected tumors within urinary diversions is crucial for accurate diagnosis and management.

## Ethics Statement

This study was approved by Nagoya University's Graduate School of Medicine's Institutional Review Board (Approval No. 2016–0474, Date: March 17, 2017).

## Consent

Patient consent was obtained through the publication of the opt‐out document.

## Conflicts of Interest

Shohei Ishida is an Editorial Board member of the International Journal of Urology and a co‐author of this article. To minimize bias, he was excluded from all editorial decision‐making related to the acceptance of this article for publication.
